# Association of rs6265 and rs2030324 Polymorphisms in Brain-Derived Neurotrophic Factor Gene with Alzheimer’s Disease: A Meta-Analysis

**DOI:** 10.1371/journal.pone.0094961

**Published:** 2014-04-14

**Authors:** Yan Lin, Shuo Cheng, Zhutian Xie, Dongfeng Zhang

**Affiliations:** Department of Epidemiology and Health Statistics, Medical College of Qingdao University, Qingdao, Shandong, People’s Republic of China; Indiana University Bloomington, United States of America

## Abstract

**Background:**

The association between polymorphisms rs6265 and rs2030324 in brain-derived neurotrophic factor (*BDNF*) and Alzheimer’s disease (AD) has been widely reported, but the results remain controversial.

**Methods:**

A comprehensive search of Pubmed, Web of Science, China National Knowledge Infrastructure (CNKI), Wanfang Med Online and China Biology Medical literature database (CBM) was performed. Pooled odds ratios (ORs) with 95% confidence intervals (CIs) were calculated using fixed or random-effects models. We excluded the studies with OR>3.0 or OR<0.3 for sensitive analysis. Subgroup analysis by ethnicity, form of AD and gender was carried out. Meta-regression was conducted to explore the potential sources of between-study heterogeneity.

**Results:**

29 articles with 7548 cases and 7334 controls concerning rs6265 and 22 articles with 5796 cases and 5706 controls concerning rs2030324 were included in this meta-analysis. The combined evidence suggested rs6265 contributing significantly to the increased risk of AD in females (codominant: fixed-effects model (FEM): OR = 1.13, 95% CI = 1.04–1.23; dominant: FEM: OR = 1.17, 95% CI = 1.05–1.31), especially for Caucasian females (codominant: FEM: OR = 1.18, 95% CI = 1.03–1.34; dominant: FEM: OR = 1.18, 95% CI = 1.01–1.37) and female late-onset Alzheimer’s disease (LOAD) patients (codominant: FEM: OR = 1.22, 95% CI = 1.05–1.41; dominant: FEM: OR = 1.23, 95% CI = 1.03–1.46). No evidence indicated an association between rs2030324 with AD in codominant (random-effects model (REM): OR = 1.06, 95% CI = 0.89–1.26) and dominant (REM: OR = 1.05, 95% CI = 0.86–1.27) models.

**Conclusion:**

This meta-analysis suggested A allele of rs6265 might increase the risk of AD in Caucasian females and female LOAD patients. In addition, no evidence indicated an association between rs2030324 with AD. Further studies are needed to confirm these results.

## Introduction

Alzheimer’s disease (AD) is an age-associated neurodegenerative disorder characterized by progressive decline in cognitive function, which typically begins with deterioration in memory [1]. The number of people with dementia worldwide in 2010 is estimated at 35.6 million and is projected to nearly double every 20 years to 65.7 million in 2030 and 115.4 million in 2050. AD is the most common form of dementia and possibly contributes to 60–70% of cases [2]. In the US alone, AD is related with an estimated health-care cost of US$172 billion per year [3]. The overwhelming number of AD patients, combined with the staggering economic burden, makes AD a public health problem.

The key pathological changes that observed in AD brain tissue are the accumulation of neuritic extracellular amyloid plaques and intracellular neurofibrillary tangles [4]. However, the neuropathological etiology of AD remains unclear, but are probably related with the combined interaction between gene variants and environmental factors [5]. There have been many genetic polymorphisms reported to be associated with AD, such as amyloid precursor protein *(APP)*, presenilin-1(*PSEN1*), presenilin-2(*PSEN2*) [6], apolipoprotein E (*APOE*) [7], [8] and sortilin-related receptor 1 (*SORL1*) [9]. However, as a complex disorder, the genes mentioned-above can not explain the overall genetic susceptibility and it is supposed that some other genes may participate in the development of AD.

Brain-derived neurotrophic factor (BDNF), as a member of the neurotrophic family, plays an important role in the growth, development, differentiation and regeneration of various types of neurons in the central nervous system [10]. Autopsy studies found reduced mRNA expression of BDNF in the hippocampus of patients with AD [11], which implicates the possible participation of BDNF in the pathogenesis of AD. There have been many polymorphisms studied in *BDNF* gene, such as rs11030104, rs16917204, rs7103411, rs6265 and rs2030324. However, only the last two polymorphisms have been widely studied, with no linkage disequilibrium (LD) between them. What’s more, these results are inconsistent and individual studies have relatively small power to confirm this association. For example, the G allele of rs6265 confers risk effect for AD in subjects of Japanese (OR = 1.23, 95% CI = 1.02–1.47) [12], but no significant association was found in Italians [13]. Therefore, we performed a meta-analysis to identify the association of the two polymorphisms in *BDNF* and AD susceptibility.

## Materials and Methods

### Search Strategy

A literature search was performed for available articles that were published in English or Chinese (up to November 2013) from the following databases: (1) Pubmed; (2) Web of Science; (3) China National Knowledge Infrastructure (CNKI); (4) Wanfang Med Online; (5) China Biology Medical literature database (CBM). The search used the following keywords: “Alzheimer’s disease” or “AD” and “brain-derived neurotrophic factor” or “*BDNF*” and “polymorphism” or “mutation” or “variant”. We also reviewed the references of included articles to identify additional articles not captured by our database searches.

### Inclusion Criteria

Two investigators reviewed all relevant studies independently to determine whether an individual study was eligible for inclusion. If the two investigators disagreed about the eligibility of a study, a senior researcher was invited to the discussion. The inclusion criteria were as follows: (1) case-control or cohort study published as an original study to evaluate the association between rs6265 and rs2030324 polymorphisms in *BDNF* gene and AD susceptibility; (2) AD were diagnosed according to NINCDS–ADRDA criteria, DSM-IV criteria or CERAD criteria; (3) genotype frequencies were reported in the articles or could be calculated or ORs and 95% CIs can be obtained; (4) the genotype frequencies of controls are consistent with Hardy-Weinberg equilibrium (HWE); (5) if one study from the same population had been published more than once, we choose the most recent or complete one; (6) English or Chinese language articles were included.

### Data Extraction

Two investigators extracted the data independently and reached a consensus on all items. Information extracted from each study was as follows: first author, publication year, country, ethnicity of studied population, diagnostic criteria for cases, sample size, genotype distributions, mean age and the percentage of male.

### Statistical Analysis

The chi-square (χ^2^) analysis was used to test deviation from Hardy-Weinberg equilibrium (HWE) for the rs6265 and rs2030324 genotype distribution of *BDNF* gene in control groups, and P<0.05 was considered as departure from HWE. Pooled measure was used as the inverse variance weighted mean of the logarithm of odds ratio (OR) with 95% confidence intervals (CI) to evaluate the strength of the association of rs6265 and rs2030324 in *BDNF* gene with risk of AD. We conducted analysis for each polymorphism considering dominant (AA+GA vs. GG for rs6265, TT+CT vs. CC for rs2030324), recessive (AA vs. GA+GG for rs6265, TT vs. CT+CC for rs2030324) and codominant (A vs. G for rs6265, T vs. C for rs2030324) models, respectively. I^2^ of Higgins and Thompson was used to describe heterogeneity among studies [14]. The random- effects model (REM) was adopted if significant heterogeneity (I^2^>50%) was found; otherwise, the fixed-effects model (FEM) was adopted, and Mantel-Haenszel was used to assess the fixed effects. For rs6265 and rs2030324, meta-regression with restricted maximum likelihood estimation was performed to explore the potentially important covariates that might exert substantial impacts on between-study heterogeneity. Specific genetic variants causally associated with common diseases would have small effects (risk ratios mostly <2.0) [15], [16]. Therefore, for sensitive analysis, we excluded the studies with OR>3.0 or OR<0.3 for both of the two polymorphisms (rs6265 and rs2030324) to control the impact of outlier values resulting from low cell counts within each single study on the pooled effect. Moreover, the ‘leave one out’ sensitive analysis was performed using I^2^>50% as the criteria to evaluate the key studies with substantial impact on between-study heterogeneity [17]. When heterogeneity was observed, subgroup analysis was also carried out. Publication bias was evaluated by Harbord’s test. An influence analysis was performed to describe how robust the pooled estimator is to removal of individual studies. If the point estimate of its omitted analysis lies outside the 95% CI of the combined analysis, the individual study is suspected of excessive influence [18]. All the statistical analyses were conducted using the STATA version 10 (Stata Corporation, College Station, TX, USA). Two-tailed P≤0.05 was considered as statistically significant.

## Results

### Literature Search and Study Characteristics

**Figure 1:**
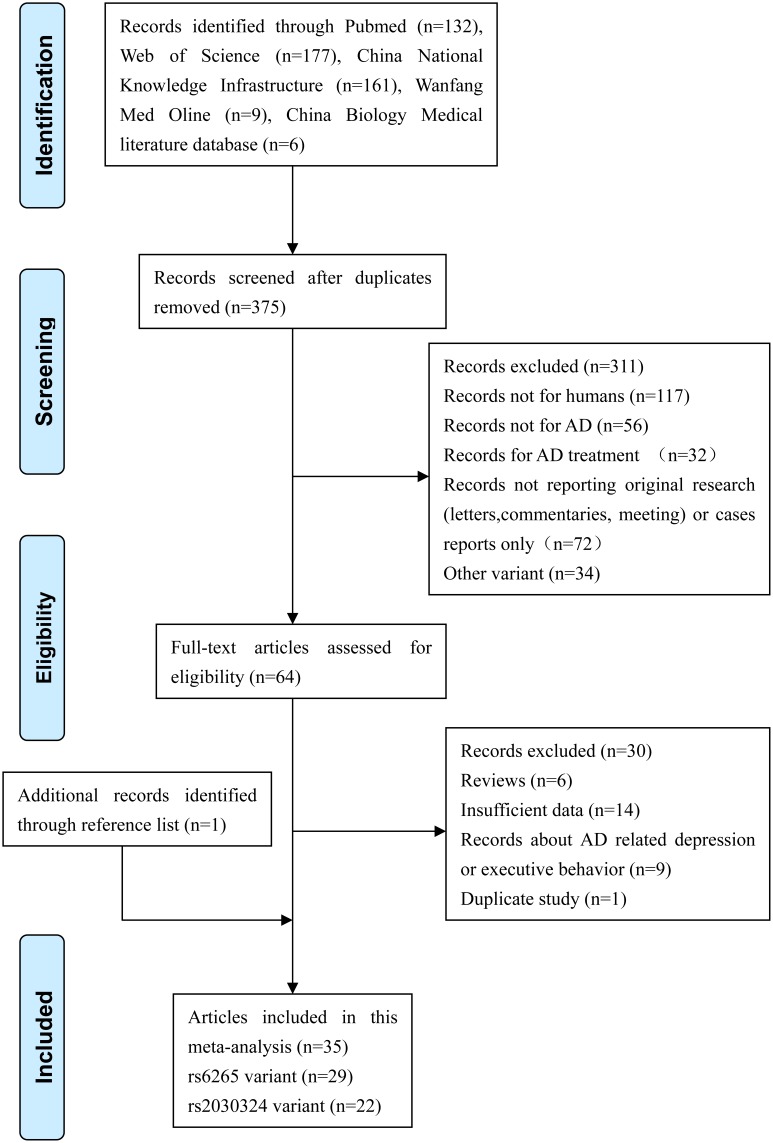
Flow diagram of literature search.

The search strategy identified 309 articles from English databases (177 articles from Web of Science, 132 articles from Pubmed), 176 articles from Chinese databases (161 articles from China National Knowledge Infrastructure, 9 articles from Wanfang Med Online, 6 articles from China Biology Medical literature database). 64 articles were reviewed in full-text. Furthermore, we excluded 30 articles according to the inclusion criteria, and obtained 1 additional article through references review. Finally, 35 articles were included in this meta-analysis. Figure 1 showed the flow diagram of literature search.

For rs6265, data from 29 published articles [12], [13], [19]–[45] with 32 studies were used including 7548 cases and 7334 controls. For rs2030324, 22 articles[12], [13], [22], [25], [26], [29], [31]–[36], [38]–[40], [43], [46]–[51] with 23 studies including 5796 cases and 5706 controls were used. The ethnicity of the studies included Caucasian, Asian, African and others. All articles were case-control studies. The detailed characteristics of the two polymorphisms are showed in Table 1, Table 2, Table 3 and Table S1 in File S1.

**Table 1:** Main characteristics of BDNF gene rs6265 polymorphism genotype distributions in studies included in this meta-analysis.

Author	Year	Country	Ethnicity	Diagnostic criteria	Mean age (case/control)	Number (case/control)	Genotypes GG/GA/AA		Allele frequency G/A		P for HWE
							AD	control	AD	control	
Boiocchi et al. [13]	2013	Italy	Caucasian	NINCDS-ADRDA	75/57	191/408	113/63/15	231/150/27	289/93	612/204	0.69
Sonali et al. [19]	2013	India	Asian	DSM-IV	64.9/64.9	57/63	3/32/22	12/23/28	38/76	47/79	0.10
Ou et al. [20]	2012	China	Asian	NINCDS-ADRDA	78.66/78.71	58/52	16/28/14	14/25/13	60/56	53/51	0.79
Borroni et al. [21]	2012	Italy	Caucasian	DSM-IV	77.6/64.2	234/162	128/87/19	89/63/10	343/125	241/83	1.00
Fukumoto et al. [22]	2010	Japan	Asian	NINCDS-ADRDA	73.5/67.1	657/525	218/319/120	197/249/79	755/559	643/407	1.00
Qi et al. [23]	2009	China	Asian	NINCDS–ADRDA	86.8/86.3	80/86	27/32/21	27/48/11	86/74	102/70	0.18
Feher et al. [24]	2009	Hungary	Caucasian	NINCDS–ADRDA	73.7/71.7	160/164	94/56/10	52/79/33	244/76	183/145	0.75
Qian et al. [25]	2008	China	Asian	NINCDS–ADRDA	72.89/72.77	105/105	28/52/25	25/56/24	108/102	106/104	0.56
Cozza et al. [26]	2008	Italy	Caucasian	DSM-IV	Na/64.2	251/97	152/84/15	60/33/4	388/114	153/41	1.00
Yu et al. [27]	2008	China	Asian	NINCDS-ADRDA	73.25/68.82	99/99	31/41/27	28/51/20	103/95	107/91	0.84
He et al. [28]	2007	China	Asian	NINCDS-ADRDA	75.7/70.1	513/575	155/245/113	165/285/125	555/471	615/535	0.93
Huang et al. [29]	2007	America	Caucasian	NINCDS-ADRDA	Na/72	220/128	150/66/4	98/25/5	366/74	221/35	0.06
Forero et al. [30]	2006	Colombia	Mixed	NINCDS–ADRDA	73.3/71.8	101/168	72/27/2	131/34/3	171/31	296/40	0.71
Zhang et al. [31]	2006	America	Caucasian	NINCDS-ADRDA	69.1/37.5	295/250	178/108/9	166/74/10	464/126	406/94	0.68
Tsai et al. [32]	2006	China	Asian	NINCDS–ADRDA	74.9/73.7	175/189	43/92/40	64/95/30	178/172	223/155	0.66
Akatsu et al. [33]	2006	Japan	Asian	CERAD	83.5/81.6	95/108	25/58/12	35/53/20	108/82	123/93	1.00
Saarela et al. [34] *	2006	Finland	Caucasian	NINCDS-ADRDA	Na/79	97/101	62/32/3	81/17/3	156/38	179/23	0.11
Lee et al. [35]	2005	America	Unknown	NINCDS–ADRDA	80.4/75	95/70	45/47/3	32/30/8	137/53	94/46	0.79
Desai et al. [36] *	2005	America	Caucasian	NINCDS–ADRDA	Na/75.7	995/671	662/299/34	456/197/18	1623/367	1109/233	0.69
Desai et al. [36] *	2005	America	African	NINCDS–ADRDA	Na/70.49	64/45	59/5/0	42/3/0	123/5	87/3	1.00
Bian et al. [37]	2005	China	Asian	NINCDS–ADRDA	Na/70.2	203/239	49/113/41	73/115/51	211/195	261/217	0.70
Vepsalainen et al. [38]	2005	Finland	Caucasian	NINCDS–ADRDA	Na/70	375/460	280/87/8	342/109/9	647/103	793/127	0.85
Matsushita et al. [12]	2005	Japan	Asian	NINCDS–ADRDA	76.1/75.2	487/471	171/247/69	150/223/98	589/385	523/419	0.41
Nishimura et al. [39]	2005	Japan	Asian	NINCDS-ADRDA	77.4/68.4	172/275	61/85/26	88/140/47	207/137	316/234	0.54
Bodner et al. [40]	2005	America	Caucasian	NINCDS-ADRDA	83/81	256/194	163/85/8	126/62/6	411/101	314/74	0.82
Li et al. [41] *	2005	England	Caucasian	NINCDS-ADRDA	Na/Na	359/396	239/105/15	269/114/13	583/135	652/140	0.86
Li et al. [41] *	2005	America	Caucasian	NINCDS-ADRDA	Na/Na	188/361	109/73/6	235/110/16	291/85	580/142	0.51
Li et al. [41] *	2005	America	Caucasian	NINCDS-ADRDA	Na/Na	388/349	251/126/11	237/105/7	628/148	579/119	0.34
Nacmias et al. [42]	2004	Italy	Caucasian	NINCDS–ADRDA	72.2/72.9	83/97	48/29/6	55/38/4	125/41	148/46	0.58
Bagnoli et al. [43]	2004	Italy	Caucasian	NINCDS-ADRDA	71.1/72.9	128/97	62/60/6	55/38/4	184/72	148/46	0.58
Combarros et al. [44]	2004	Spain	Caucasian	NINCDS-ADRDA	75.3/79.9	237/218	149/78/10	143/67/8	376/98	353/83	1.00
Ventriglia et al. [45]	2002	Italy	Caucasian	NINCDS-ADRDA	72/Na	130/111	85/33/12	54/48/9	203/57	156/66	0.82

* The cases of these studies are late-onset Alzheimer’s disease (LOAD); Abbreviations: Na, not available; HWE, Hardy-Weinberg equilibrium. NINCDS-ADRDA, National Institute of Neurological and Communicative Disorders and Stroke-Alzheimer’s Disease and Related Disorders Association criteria; DSM-IV, Diagnostic and Statistical Manual of Mental Disorders (Fourth Edition); CERAD, Consortium to Establish a Registry for Alzheimer’s disease.

**Table 2:** Genotype and allele distribution for rs6265 polymorphism in female and other subgroups.

Author	Year	Country	Ethnicity	Mean age (case/control)	Genotypes GG/GA/AA		Allele frequency (G/A)		Genotypes GG/GA/AA		Genotypes GG/GA/AA		Allele frequency (G/A)	
					AD(female)	control	AD(female)	control	AD(male)	control	LOAD	control	LOAD	control
Boiocchi et al. [13]	2013	Italy	Caucasian	75/57	69/42/9	130/81/14	180/60	341/109	109/33	271/95	Na	Na	Na	Na
Ou et al. [20]	2012	China	Asian	78.66/78.71	7/6/5	4/7/5	20/16	15/17	219/171	251/235	Na	Na	Na	Na
Fukumoto et al. [22]	2010	Japan	Asian	73.5/67.1	142/205/80	122/143/40	489/365	387/223	40/40	38/34	Na	Na	Na	Na
Yu et al [27]	2008	China	Asian	73.25/68.82	13/11/13	11/27/11	37/37	49/49	47/21	40/24	Na	Na	Na	Na
He et al. [28]	2007	China	Asian	75.7/70.1	92/152/74	97/170/65	336/300	364/300	34/16	48/24	131/189/93	128/208/94	451/375	464/396
Forero et al. [30]	2006	Colombia	Mixed	73.3/71.8	51/20/2	90/23/2	122/24	203/27	49/7	93/13	Na	Na	Na	Na
Tsai et al. [32]	2006	China	Asian	74.9/73.7	19/50/15	33/50/18	88/80	116/86	530/128	420/100	Na	Na	Na	Na
Akatsu et al. [33]	2006	Japan	Asian	83.5/81.6	16/36/6	30/42/14	68/48	102/70	35/1	23/1	Na	Na	Na	Na
Saarela et al. [34] *	2006	Finland	Caucasian	Na/79	45/21/2	46/10/0	111/25	102/10	104/86	142/126	62/32/3	81/17/3	156/38	179/23
Lee et al. [35]	2005	America	Unknown	80.4/75	31/28/2	20/14/4	90/32	54/22	185/109	161/139	Na	Na	Na	Na
Desai et al. [36] *	2005	America	Caucasian	Na/75.7	449/201/19	287/115/9	1099/239	689/133	90/92	107/69	662/299/34	456/197/18	1623/367	1109/233
Desai et al. [36] *	2005	America	African	Na/70.49	42/4/0	31/2/0	88/4	64/2	40/34	21/23	59/5/0	42/3/0	123/5	87/3
Bian et al. [37]	2005	China	Asian	Na/70.2	20/67/21	36/47/22	107/109	119/91	45/13	77/13	34/73/27	58/90/42	141/127	206/174
Matsushita et al. [12]	2005	Japan	Asian	76.1/75.2	117/170/53	104/154/63	404/276	362/280	66/58	58/42	137/195/54	150/223/98	469/303	523/419
Li et al. [41] *	2005	England	Caucasian	Na/Na	178/73/14	192/73/5	429/101	457/83	266/194	256/184	239/105/15	269/114/13	583/135	652/140
Li et al. [41] *	2005	America	Caucasian	Na/Na	51/32/4	150/67/9	134/40	367/85	115/37	99/27	109/73/6	235/110/16	291/85	580/142
Li et al. [41] *	2005	America	Caucasian	Na/Na	163/81/4	150/60/5	407/89	360/70	118/26	140/38	251/126/11	237/105/7	628/148	579/119
Nacmias et al. [42]	2004	Italy	Caucasian	72.2/72.9	36/19/3	39/22/0	91/25	100/22	146/42	201/51	Na	Na	Na	Na
Combarros et al. [44]	2004	Spain	Caucasian	75.3/79.9	107/47/7	105/44/6	261/61	254/56	221/59	219/49	Na	Na	Na	Na

* The cases of these studies are late-onset Alzheimer’s disease (LOAD). Abbreviations: Na, not available.

**Table 3:** Main characteristics of BDNF gene rs2030324 polymorphism genotype and allele distributions in studies included in this meta-analysis.

Author	Year	Country	Ethnicity	Diagnostic criteria	Mean age (case/control)	Number (case/control)	Genotypes CC/CT/TT		Allele frequency C/T		P for HWE
							AD	control	AD	control	
Boiocchi et al. [13]	2013	Italy	Caucasian	NINCDS-ADRDA	75/57	192/384	55/93/44	103/192/89	203/181	398/370	1.00
Cousin et al. [46]	2011	France	Caucasian	NINCDS-ADRDA	Na/66.2	425/470	370/54/1	419/50/1	794/56	888/52	1.00
Fukumoto et al. [22]	2010	Japan	Asian	NINCDS-ADRDA	73.5/67.1	657/525	611/45/1	490/34/1	1267/47	1014/36	0.46
Hou et al. [47] *	2009	China	Asian	NINCDS-ADRDA	79.21/76.07	203/138	172/31/0	117/21/0	375/31	255/21	1.00
Qian et al. [25]	2008	China	Asian	NINCDS–ADRDA	72.89/72.77	105/105	104/1/0	96/8/1	209/1	200/10	0.20
Cozza et al. [26]	2008	Italy	Caucasian	DSM-IV	Na/64.2	251/97	212/35/4	80/15/2	459/43	175/19	0.22
Huang et al. [29]	2007	America	Caucasian	NINCDS-ADRDA	Na/72	220/128	202/16/2	113/15/0	420/20	241/15	1.00
Zhang et al. [31]	2006	America	Caucasian	NINCDS-ADRDA	69.1/37.5	295/250	271/22/2	220/30/0	564/26	470/30	1.00
Tsai et al. [32]	2006	China	Asian	NINCDS–ADRDA	74.9/73.7	175/189	151/24/0	167/20/2	326/24	354/24	0.16
Akatsu et al. [33]	2006	Japan	Asian	CERAD	83.5/81.6	95/108	89/6/0	101/7/0	184/6	209/7	1.00
Saarela et al. [34] *	2006	Finland	Caucasian	NINCDS-ADRDA	Na/79	97/101	88/9/0	81/19/1	185/9	181/21	1.00
Lee et al. [35]	2005	American	Caucasian	NINCDS–ADRDA	80.4/75	106/73	102/4/0	66/7/0	208/4	139/7	1.00
Desai et al. [36] *	2005	American	Caucasian	NINCDS–ADRDA	Na/75.7	719/523	629/86/4	454/69/0	1344/94	977/69	0.15
Desai et al. [36] *	2005	American	African	NINCDS–ADRDA	Na/70.49	58/42	54/4/0	38/4/0	112/4	80/4	1.00
Olin et al. [48]	2005	American	Caucasian	Na	77.7/66	212/202	173/36/3	189/13/0	382/42	391/13	1.00
Vepsalainen et al. [38]	2005	Finland	Caucasian	NINCDS–ADRDA	Na/70	375/460	90/199/86	124/239/97	379/371	487/433	0.40
Matsushita et al. [12]	2005	Japan	Asian	NINCDS–ADRDA	76.1/75.2	487/471	457/30/0	438/33/0	944/30	909/33	1.00
Nishimura et al. [39]	2005	Japan	Asian	NINCDS-ADRDA	77.4/68.4	172/275	154/18/0	264/11/0	326/18	539/11	1.00
Bodner et al. [40]	2005	American	Caucasian	NINCDS-ADRDA	83/81	256/194	230/26/0	175/19/0	486/26	369/19	1.00
Nishimura et al. [49] *	2004	Brazil	Caucasian	NINCDS–ADRDA	68.7/72.3	188/188	175/13/0	170/17/1	363/13	357/19	0.38
Bagnoli et al. [43]	2004	Italy	Caucasian	NINCDS-ADRDA	71.1/72.9	128/97	113/14/1	83/14/0	240/16	180/14	1.00
Riemenschneider et al. [50]	2002	German	Caucasian	NINCDS-ADRDA	69.3/65.6	210/188	185/24/1	175/13/0	394/26	363/13	1.00
Kunugi et al. [51]	2001	Japan	Asian	NINCDS-ADRDA	74/55	170/498	150/19/1	477/21/0	319/21	975/21	1.00

* The cases of these studies are late-onset Alzheimer’s disease (LOAD); Abbreviations: HWE, Hardy-Weinberg equilibrium. NINCDS-ADRDA, National Institute of Neurological and Communicative Disorders and Stroke-Alzheimer’s Disease and Related Disorders Association criteria; DSM-IV, Diagnostic and Statistical Manual of Mental Disorders (Fourth Edition); CERAD, Consortium to Establish a Registry for Alzheimer’s disease.

### Influence Analysis and Publication Bias

For both of the two polymorphisms, no individual study has excessive influence on the pooled effect in any of dominant, recessive and codominant models (data not shown). Harbord’s test showed no publication bias for both two polymorphisms.

### Quantitative Synthesis

#### Overall analysis for rs6265 and rs2030324

**Figure 2:**
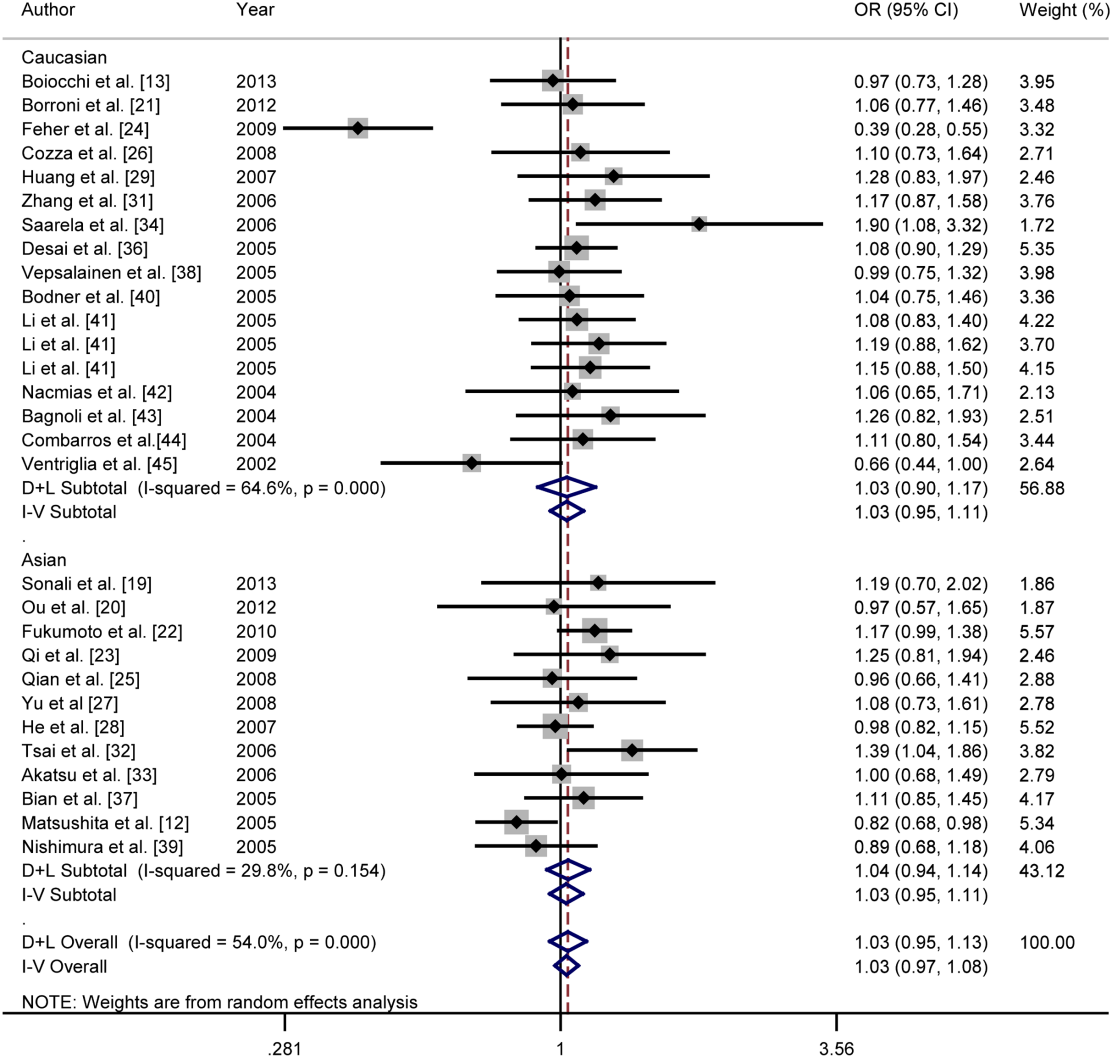
Forest plots of relationship between BDNF gene rs6265 polymorphism and AD risk in codominant model (A vs. G) for overall analysis. White diamond donates the pooled OR. Black squares indicate the OR in each study, with square sizes inversely proportional to the standard error of the OR. Horizontal lines represent 95% CIs.

For overall analysis, no significant association was found between A allele and risk of AD in codominant (REM: OR = 1.03, 95% CI = 0.95–1.12), dominant (REM: OR = 1.06, 95% CI = 0.95–1.18) and recessive model (FEM: OR = 1.00, 95% CI = 0.89–1.12) for rs6265 polymorphism. No evidence indicated an association between rs2030324 with AD in codominant (REM: OR = 1.06, 95% CI = 0.89–1.26), dominant (REM: OR = 1.05, 95% CI = 0.86–1.27) and recessive (FEM: OR = 1.09, 95% CI = 0.85–1.39) models. The results for overall analysis are showed in Table 4. Figure 2 presented the forest plot of ORs in codominant model (A vs. G) in overall analysis for rs6265.

**Table 4:** The overall pooled measure on the relation of BDNF gene rs6265 and rs2030324 polymorphism with AD.

Loci	Inherited model	All included articles					After excluding articles with OR>3.0 or OR<0.3					
		Number	REM Pooled OR (95% CI)	FEM Pooled OR (95% CI)	Q-value	I^2^ (%)	Number	REM Pooled OR (95% CI)	FEM Pooled OR (95% CI)	Q-value	I^2^(%)	Articles Excluded
rs6265	Codominant	7548/7334	1.03(0.95–1.12)	1.03(0.97–1.08)	63.14	50.9	7548/7334	-	-	-	-	-
	Dominant	7548/7334	1.06(0.95–1.18)	1.05(0.98–1.13)	63.92	51.5	7491/7271	1.05(0.94–1.16)	1.05(0.98–1.12)	59.64	49.7	[19]
	Recessive^a^	7484/7289	1.00(0.86–1.17)	1.00(0.89–1.12)	42.74	29.8	7313/7100	1.04(0.93–1.17)	1.04(0.93–1.17)	28.00	0	[24], [35]
rs2030324	Codominant	5796/5706	1.06(0.89–1.26)	1.06(0.96–1.17)	52.13	57.8	5309/4901	0.99(0.88–1.12)	1.01(0.91–1.12)	22.49	15.5	[25], [48], [51]
	Dominant	5796/5706	1.05(0.86–1.27)	1.06(0.94–1.19)	51.64	57.4	5309/4901	0.98(0.85–1.13)	0.99(0.88–1.12)	24.11	21.2	[25], [48], [51]
	Recessive^b^	4419/4405	1.09(0.85–1.39)	1.09(0.85–1.39)	15	0	2848/2743	1.05(0.82–1.34)	1.05(0.82–1.34)	10	0	[31], [32], [36], [48], [51]

a One study about African (Desaia et al)for recessive model was not sufficient to calculated pooled OR.

b Seven studies (Hou et al. Akatsu et al. Lee et al. Desaia et al. Matsushita et al. Masataka et al. Bodner et al.) for recessive model were not sufficient to calculated pooled OR.

Abbreviations: AD, Alzheimer’s disease; FEM, fixed-effects model; REM, random-effects model.

rs6265: Codominant model, A vs. G; Dominant model, AA+GA vs. GG; Recessive model, AA vs. GA+GG.

rs2030324: Codominant model, T vs C; Dominant model, TT+CT vs. CC; Recessive model, TT vs. CT+CC.

Evidence for heterogeneity (I^2^>50%) was found in codominant and dominant models considering the association of rs6265 and rs2030324 polymorphisms with AD. Univariate meta-regression with the covariates of publication year, ethnicity, diagnostic criteria, form of AD, age (ratio of mean age in case group to that in control group) and gender (ratio of male percent in case group to that in control group) for the above-mentioned polymorphisms showed that no covariates have significant effect on between-study heterogeneity.

### Subgroup Analysis for Both Two Polymorphisms

**Figure 3:**
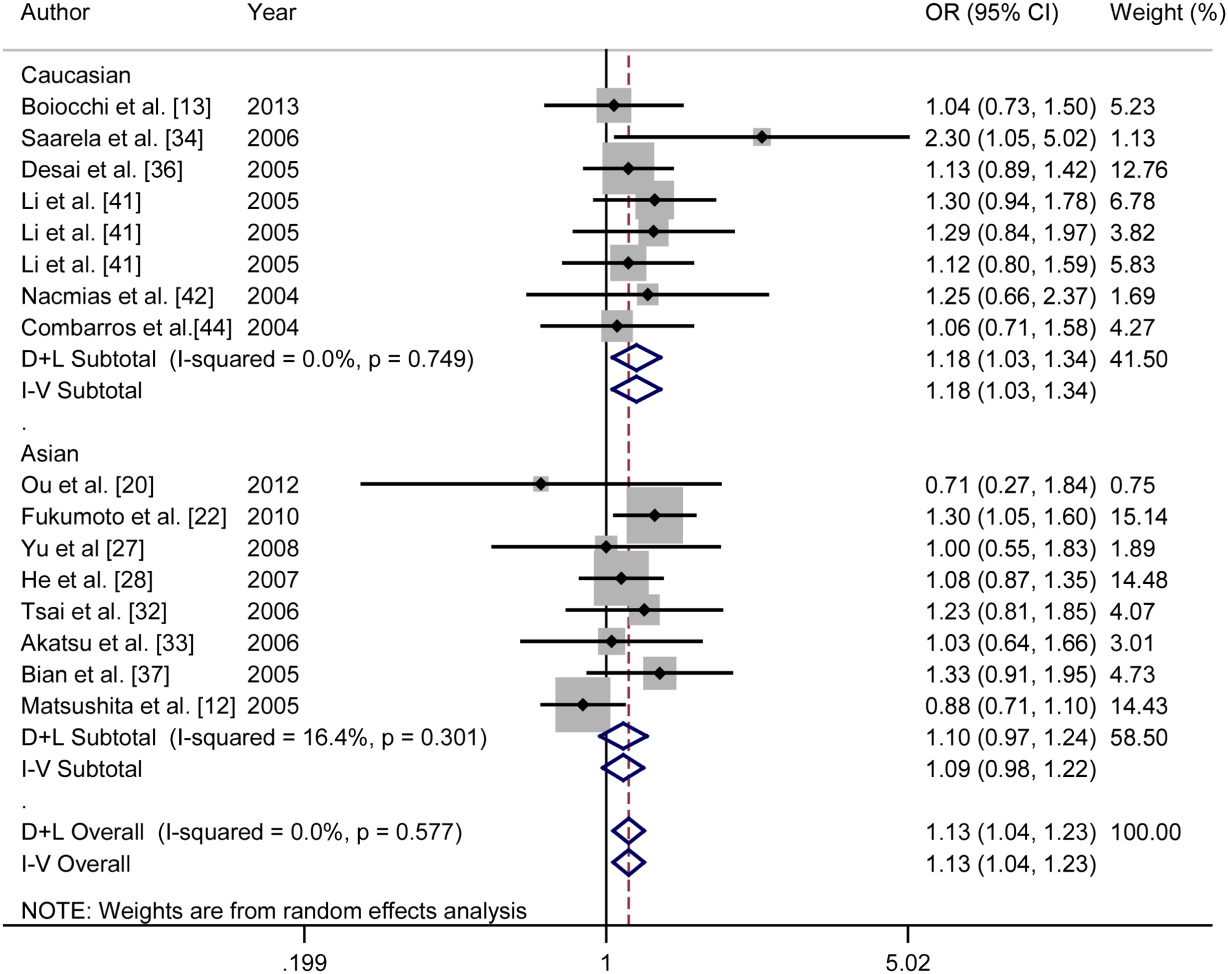
Forest plots of relationship between BDNF gene rs6265 polymorphism and AD risk in codominant model (A vs. G) for female group. White diamond donates the pooled OR. Black squares indicate the OR in each study, with square sizes inversely proportional to the standard error of the OR. Horizontal lines represent 95% CIs.

In the stratified analysis by ethnicity, no evidence indicated the association between rs6265 and AD susceptibility both for Caucasians and Asians in codominant (Caucasian: REM: OR = 1.03, 95% CI = 0.90–1.17; Asian: FEM: OR = 1.03, 95% CI = 0.95–1.11), dominant (Caucasian: REM: OR = 1.04, 95% CI = 0.88–1.22; Asian: FEM: OR = 1.05, 95% CI = 0.94–1.18) and recessive (Caucasian: FEM: OR = 1.01, 95% CI = 0.81–1.25; Asian: FEM: OR = 1.01, 95% CI = 0.88–1.15) models. After stratified by gender, a statistical significant association between rs6265 and risk of AD was observed for females in codominant (FEM: OR = 1.13, 95% CI = 1.04–1.23) and dominant model (FEM: OR = 1.17, 95% CI = 1.05–1.31), but not in recessive model (FEM: OR = 1.13, 95% CI = 0.95–1.35). However, no association was detected for males in codominant model (FEM: OR = 0.98, 95% CI = 0.88–1.08), dominant model (OR = 1.01, 95% CI = 0.88–1.16) and recessive model (FEM: OR = 0.91, 95% CI = 0.74–1.12). When stratified by ethnicity in females, for Caucasian females, the A allele was found contributing significantly to the increased risk of AD in codominant model (FEM: OR = 1.18, 95% CI = 1.03–1.34) and dominant model (FEM: OR = 1.18, 95% CI = 1.01–1.37), not in recessive model (FEM: OR = 1.40, 95% CI = 0.94–2.10). With regard to Asian females, no association was detected in codominant model (FEM: OR = 1.09, 95% CI = 0.98–1.22), dominant model (FEM: OR = 1.15, 95% CI = 0.98–1.36) and recessive model (FEM: OR = 1.09, 95% CI = 0.90–1.33). When stratified by gender in LOAD patients, the A allele was observed significantly associated with AD in female LOAD patients in codominant model (FEM: OR = 1.22, 95% CI = 1.05–1.41) and dominant model (FEM: OR = 1.23, 95% CI = 1.03–1.46), but not in recessive model (FEM: OR = 1.47, 95% CI = 0.88–2.44). However, no association was found in male LOAD patients in any of the above-mentioned models. Figure 3 presented the forest plot of ORs in codominant model (A vs. G) in female group for rs6265.

For rs2030324, in the subgroup analysis, for Caucasian, no association was found in codominant model (REM: OR = 1.00, 95% CI = 0.82–1.21), dominant model (REM: OR = 0.97, 95% CI = 0.78–1.22) and recessive model (FEM: OR = 1.10, 95% CI = 0.86–1.40). With regard to Asians, there was no association between T allele and AD susceptibility in codominant model (REM: OR = 1.22, 95% CI = 0.80–1.85), dominant model (REM: OR = 1.24, 95% CI = 0.82–1.89) and recessive model (FEM: OR = 0.81, 95% CI = 0.18–3.71). When stratified by gender, there was still no association between rs2030324 and AD both for females and males in codominant model (Female: FEM OR = 0.97, 95% CI = 0.75–1.26; Male: FEM OR = 1.02, 95% CI = 0.74–1.40), dominant model (Female: FEM: OR = 1.02, 95% CI = 0.74–1.40; Male: OR = 0.87, 95% CI = 0.56–1.34). The pooled results are summarized in Table 5 and Table S2 and Table S3 in File S1.

**Table 5:** Subgroup analysis on the relation of BDNF gene rs6265 and rs2030324polymorphism with AD in codominant model.

Loci	Data	Type	Inherited model	All included articles					After excluding articles with OR>3.0 or OR<0.3					
				Number	REM Pooled OR (95% CI)	FEM Pooled OR (95% CI)	Q-value	I^2^ (%)	Number	REM PooledOR (95% CI)	FEM PooledOR (95% CI)	Q-value	I^2^ (%)	Articles Excluded
rs6265	Ethnicity	Caucasian	Codominant	4587/4264	1.03(0.90–1.17)	1.03(0.95–1.11)	45.2	64.6	-	-	-		-	-
		Asian	Codominant	2701/2787	1.04(0.94–1.14)	1.03(0.95–1.11)	15.67	29.8	-	-	-	-	-	-
	Gender	**Female**	**Codominant**	**3246/3120**	**1.13(1.04–1.23)**	**1.13(1.04–1.23)**	**18**	**0**	-	-	-	-	-	-
		Male	Codominant	1813/1976	0.98(0.88–1.08)	0.98(0.88–1.08)	18	0	-	-	-	-	-	-
	Form	EOAD	Codominant	270/665	0.96(0.78–1.19)	0.96(0.78–1.19)	2	0	-	-	-		-	-
		LOAD	Codominant	3024/3014	1.05(0.93–1.18)	1.03(0.94–1.12)	12.97	38.3	-	-	-		-	-
	Female	**Caucasian**	**Codominant**	**1676/1619**	**1.18(1.03–1.34)**	**1.18(1.03–1.34)**	**7**	**0**	-	-	-	-	-	-
		Asian	Codominant	1390/1315	1.10(0.97–1.24)	1.09(0.98–1.22)	8.37	16.4	-	-	-	-	-	-
	Male	Caucasian	Codominant	836/936	1.04(0.88–1.23)	1.04(0.88–1.23)	7	0	-	-	-	-	-	-
		Asian	Codominant	897/943	0.97(0.80–1.16)	0.95(0.83–1.08)	12.13	42.3	-	-	-	-	-	-
	LOAD	**Female**	**Codominant**	**1383/1211**	**1.22(1.05–1.41)**	**1.22(1.05–1.41)**	**5**	**0**	-	-	-	-	-	-
		Male	Codominant	682/666	1.07(0.88–1.30)	1.07(0.88–1.30)	5	0		-	-	-	-	-
rs2030324	Ethnicity	Caucasian	Codominant	3674/3355	1.00(0.82–1.21)	1.03(0.92–1.15)	28.63	54.6	3462/3153	0.96(0.84–1.11)	0.99(0.89–1.11)	15.35	21.8	[48]
		Asian	Codominant	2064/2309	1.22(0.80–1.85)	1.22(0.98–1.52)	21.41	67.3	1789/1706	1.11(0.85–1.47)	1.10(0.86–1.39)	6.34	21.1	[25], [51]
	Form	EOAD	Codominant	303/1359	1.39(0.66–2.90)	1.55(0.95–2.53)	6.3	52.4	-	-	-	-	-	-
		LOAD	Codominant	2038/2351	1.21(0.77–1.89)	1.18(0.97–1.43)	36.2	77.9	1760/1651	0.91(0.74–1.13)	0.91(0.74–1.13)	6	0	[48], [51]
	Gender	Female	Codominant	696/603	0.97(0.75–1.26)	0.97(0.75–1.26)	2	0	-	-	-	-	-	-
		Male	Codominant	356/444	1.02(0.74–1.40)	1.02(0.74–1.40)	2	0	-	-	-		-	-

Abbreviations: FEM, fixed-effects model; REM, random-effects model. EOAD, early-onset Alzheimer’s Disease; LOAD, late-onset Alzheimer’s Disease.

rs6265: Codominant model, A vs. G; rs2030324: Codominant model, T vs C.

### Sensitivity Analysis

After excluding articles with OR>3.0 or OR<0.3, low heterogeneity (I^2^<50%) was found in the codominant, dominant and recessive models, and the results for both two polymorphisms are consistent with the ones without sensitive analysis. Furthermore, the “leave one out” analysis was carried out when significant heterogeneity (I^2^>50%) was observed. After excluded key articles with substantial impact on between-study heterogeneity, the data showed rs6265 confers a risk effect for Caucasians in codominant model (FEM: OR = 1.08, 95% CI = 1.00–1.17, P = 0.042). With respect to rs2030324, the result indicated T allele was associated with EOAD patients in codominant (FEM: OR = 2.02, 95% CI = 1.18–3.47) and dominant model (FEM: OR = 2.01, 95% CI = 1.15–3.52).

## Discussion

To our knowledge, many case-control studies have been carried out to investigate the role of *BDNF* gene in the development of AD. However, these results remained controversial. 29 articles concerning rs6265 and 22 articles concerning rs2030324 were included in our meta-analysis. With respect to rs2030324, there was no evidence for an association with AD. What’s more, the combined evidence suggested that rs6265 was not associated with AD for overall analysis. After stratified by ethnicity in females, our data indicated rs6265 lead to the increased risk of AD in Caucasian females, but not for Asians. When stratified by gender in LOAD patients, the A allele was found contributing significantly to the increased risk of AD in female LOAD patients. The results of our study were not consistent with the previous meta-analysis conducted by Fukumoto et al. [22] in 2010 including 16 studies revealing a gender-related association between rs6265 polymorphism and AD susceptibility. The reason might be that the quantitative assessments in our study were based on a larger sample size and we have performed a detailed subgroup analysis.

The Met66-BDNF protein has been shown to be associated with reduced transport of BDNF from the Golgi region to appropriate secretory granules in neurons, compared with the Val66-BDNF protein [52]. Moreover, the A allele of rs6265 was related with poorer episodic memory, abnormal hippocampal activation, and lower hippocampal n-acetyl aspartate (NAA) in human subjects [53]. Epidemiological studies showed higher incidence and prevalence of AD in women than in men [3], [5]. Molecular mechanisms underlying this correlation had been considered as colocalization estrogen receptors with BDNF-synthesizing neurons in the forebrain [54] and induction BDNF expression by estrogen through the estrogen response element [55]. In addition, though the two forms of AD (EOAD and LOAD) have different patterns of genetic epidemiology [4], the results of our study revealed no difference between them.

Between-study heterogeneity is common in meta-analysis for genetic association studies [56] and it is essential to explore the potential sources of between-study heterogeneity [57]. This meta-analysis also showed significant between-study heterogeneity in dominant model and codominant model for both two polymorphisms. An indeterminate number of characteristics that varied among studies could be the sources of between-study heterogeneity, such as publication year, ethnicity, diagnostic criteria, form of AD, age, and gender etc. Therefore, in order to explore the potential sources of between-study heterogeneity for rs6265 and rs2030324, meta-regression was adopted. However, the aforementioned covariates were not important contributors to this disease-effect heterogeneity. Considering that our meta-analysis showed significant heterogeneity, subgroup analyses by ethnicity (Caucasian and Asian), form of AD (EOAD and LOAD) and gender (female and male) etc. were performed to explore the sources of heterogeneity. However, between-study heterogeneity still existed in subgroups, suggesting the presence of other unknown confounders. AD is a complex multi-factorial disease and is related with the combined effects between gene variants and environmental factors. Therefore, other genetic and environment variables, as well as their possible interaction, may be potential contributors to this disease-effect unconformity. We further conducted a sensitivity analysis excluding articles with OR>3 or OR<0.3. After sensitivity analysis, low heterogeneity (I^2^<50%) was found in the codominant, dominant and recessive models, and the results are consistent with the one before heterogeneity analysis for rs6265 and rs2030324, strongly identified the stability of our results. Moreover, no publication bias was found in any of the above-mentioned models for both two polymorphisms.

The major strength of our meta-analysis is that the results were based on the large number of participators, allowing a much greater possibility of reaching definitive conclusions. Additionally, we conducted subgroup analyses to explore the potential sources of heterogeneity, and sensitivity analysis was carried out to ensure the stability of our results. However, our meta-analysis also had some limitations. Firstly, lack of the original data of included articles made it impracticable to excluded potential confounders completely, especially the confounding of age. Secondly, different diagnostic criteria may have possible influence on the diagnosis of AD.

In conclusion, this meta-analysis suggested A allele of rs6265 might increase the risk of AD in Caucasian females and female LOAD patients. In addition, no evidence indicated an association between rs2030324 with AD. Since potential biases and confounders could not be ruled out completely in this study, further studies are needed to confirm these results.

## Supporting Information

File S1 **This includes the files Search Strategy S1 and Tables S1 to S3.** Search Strategy S1. Keywords of literature search for different database. Table S1. Genotype and allele distribution for rs2030324 polymorphism in female and other subgroups. Table S2. Subgroup analysis on the relation of BDNF gene rs6265 polymorphism with AD in dominant and recessive model. Table S3. Subgroup analysis on the relation of BDNF gene rs2030324 polymorphism with AD in dominant and recessive model.
(DOC)

Checklist S1 **PRISMA checklist.**
(DOC)
