# Supplementary material for: Association of rs6265 and rs2030324 Polymorphisms in Brain-Derived Neurotrophic Factor Gene with Alzheimer’s Disease: A Meta-Analysis
Source: PLoS One. 2014 Apr 14;9(4):e94961. doi: 10.1371/journal.pone.0094961 (PMC3986375; doi:10.1371/journal.pone.0094961)
Supplement: File S1 — This includes the files Search Strategy S1 and Tables S1 to S3. Search Strategy S1. Keywords of literature search for different database. Table S1. Genotype and allele distribution for rs2030324 polymorphism in female and other subgroups. Table S2. Subgroup analysis on the relation of BDNF gene rs6265 polymorphism with AD in dominant and recessive model. Table S3. Subgroup analysis on the relation of BDNF gene rs2030324 polymorphism with AD in dominant and recessive model. (DOC) [file pone.0094961.s001.doc]

**Search Strategy S1： Keywords of literature search for different database**

The literature search was up to November 2013

**Pubmed:** “Alzheimer’s disease” or “AD” and “brain-derived neurotrophic factor” or “BDNF” and “polymorphism” or “mutation” or “variant”. We searched via full-text research and identified 132 articles.

**Web of Science:** “Alzheimer’s disease” and “brain-derived neurotrophic factor” or “BDNF” and “polymorphism” or “mutation” or “variant” (177). We searched via subject heading research and obtained 177 articles.

**China National Knowledge Infrastructure (CNKI)**：“Alzheimer’s disease”（阿尔茨海默病）and “brain-derived neurotrophic factor”（脑源性神经营养因子）and “polymorphism”（多态性）(161). We searched via full-text research and identified 161 articles.

**China Biology Medical literature database (CBM)：**“Alzheimer’s disease”（阿尔茨海默病）and “brain-derived neurotrophic factor”（脑源性神经营养因子）and “polymorphism”（多态性）(6). We searched via full-text research and identified 6 articles.

**Wan Fang Med Online:** “Alzheimer’s disease”（阿尔茨海默病）and “brain-derived neurotrophic factor”（脑源性神经营养因子）and “polymorphism”（多态性）(9). We searched via full-text research and identified 9 articles.

| **Table S1.** Genotype and allele distribution for rs2030324 polymorphism in female and other subgroups | | | | | | | | | | | | | | |  |  |
| --- | --- | --- | --- | --- | --- | --- | --- | --- | --- | --- | --- | --- | --- | --- | --- | --- |
| Author | Year | Country | Ethnicity | Mean age (case/control) | Genotypes CC/CT/TT | | Allele frequency C/T | | Genotypes CC/CT/TT | | Allele frequency C/T | | Genotypes CC/CT/TT | | Allele frequency C/T | |
|  |  |  |  |  | AD(female) | control | AD(female) | control | AD(male) | control | AD(male) | control | LOAD | control | LOAD | control |
| Boiocchi et al. [13] | 2013 | Italy | Caucasian | 75/57 | 35/63/24 | 63/95/51 | 133/111 | 221/197 | 20/30/20 | 40/97/38 | 70/70 | 177/173 | Na | Na | Na | Na |
| Fukumoto et al. [22] | 2010 | Japan | Asian | 73.5/67.1 | 396/31/0 | 285/20/0 | 273/21 | 165/13 | 46/10/0 | 41/8/0 | 102/10 | 90/8 | Na | Na | Na | Na |
| Hou et al. [47]* | 2009 | China | Asian | 79.21/76.07 | 126/21/0 | 76/13/0 | 823/31 | 590/20 | 215/14/1 | 205/14/1 | 444/16 | 424/16 | 172/31/0 | 117/21/0 | 375/31 | 255/21 |
| Saarela et al. [34]* | 2006 | Finland | Caucasian | Na/79 | Na | Na | Na | Na | Na | Na | Na | Na | 88/9/0 | 81/19/1 | 185/9 | 181/21 |
| Desai et al. [36]* | 2005 | American | Caucasian | Na/75.7 | Na | Na | Na | Na | Na | Na | Na | Na | 629/86/4 | 454/69/0 | 1344/94 | 977/69 |
| Desai et al. [36]* | 2005 | American | African | Na/70.49 | Na | Na | Na | Na | Na | Na | Na | Na | 54/4/0 | 38/4/0 | 112/4 | 80/4 |
| Nishimura et al. [49]* | 2004 | Brazil | Caucasian | 68.7/72.3 | Na | Na | Na | Na | Na | Na | Na | Na | 175/13/0 | 170/17/1 | 363/13 | 357/19 |
| Olin et al. [48] | 2005 | American | Caucasian | 77.7/66 | Na | Na | Na | Na | Na | Na | Na | Na | 126/30/3 | 189/13/0 | 282/36 | 391/13 |
| Matsushita et al. [12] | 2005 | Japan | Asian | 76.1/75.2 | Na | Na | Na | Na | Na | Na | Na | Na | 359/27/0 | 438/33/0 | 745/27 | 909/33 |
| Riemenschneider et al. [50] | 2002 | German | Caucasian | 69.3/65.6 | Na | Na | Na | Na | Na | Na | Na | Na | 99/10/0 | 175/13/0 | 208/10 | 363/13 |
| Kunugi et al. [51] | 2001 | Japan | Asian | 74/55 | Na | Na | Na | Na | Na | Na | Na | Na | 102/16/1 | 477/21/0 | 220/18 | 975/21 |

* The cases of these studies are late-onset Alzheimer's disease (LOAD). Abbreviations: Na, not available

| **Table S2.** Subgroup analysis on the relation of BDNF gene rs6265 polymorphism with AD in dominant and recessive model | | | | | | | | | | | | | |
| --- | --- | --- | --- | --- | --- | --- | --- | --- | --- | --- | --- | --- | --- |
| Data | Type | Inherited model | All included articles | | | | |  | After excluding articles with OR>3.0 or OR<0.3 | | | | |
|  |  |  | Number | REM Pooled OR (95% CI) | FEM Pooled OR (95% CI) | Q-value | I² (%) | Number | REM Pooled OR (95% CI) | FEM Pooled OR (95% CI) | Q-value | I² (%) | Articles Excluded |
| Ethnicity | Caucasian | Dominant | 4587/4264 | 1.04(0.88-1.22) | 1.04(0.95-1.14) | 46.92 | 65.9 | - | - | - |  | - | - |
|  |  | Recessive | 4587/4264 | 1.00(0.79-1.27) | 1.01(0.81-1.25) | 18.41 | 13.1 | 4424/4100 | 1.14(0.91-1.43) | 1.14(0.91-1.43) | 15.00 | 0 | [24] |
|  | Asian | Dominant | 2701/2787 | 1.06(0.91-1.24) | 1.05(0.94-1.18) | 15.74 | 30.1 | 2644/2724 | 1.04(0.92-1.19) | 1.04(0.93-1.17) | 11.44 | 12.6 | [19] |
|  |  | Recessive | 2701/2787 | 1.03(0.84-1.25) | 1.01(0.88-1.15) | 20.41 | 46.1 | - | - | - | - | - | - |
| Sex | Female | Dominant | 3246/3120 | 1.17(1.05-1.31) | 1.17(1.05-1.31) | 18 | 0 | - | - | - | - | - | - |
|  |  | Recessive ^a^ | 3200/3087 | 1.13(0.94-1.37) | 1.13(0.95-1.35) | 18.18 | 6.5 | 3013/2932 | 1.14(0.95-1.36) | 1.14(0.95-1.36) | 15 | 0 | [34,35,42] |
|  | Male | Dominant | 1813/1976 | 1.01(0.88-1.16) | 1.01(0.88-1.16) | 18 | 0 | - | - | - | - | - | - |
|  |  | Recessive ^a^ | 1795/1964 | 0.92(0.70-1.22) | 0.91(0.74-1.12) | 24.05 | 29.3 | 1520/1664 | 0.93(0.72-1.21) | 0.92(0.74-1.13) | 16.65 | 21.9 | [34,35,41] |
| Form | EOAD | Dominant | 270/665 | 1.06(0.76-1.47) | 1.06(0.76-1.47) | 2 | 0 | - | - | - |  | - | - |
|  |  | Recessive | 270/665 | 0.83(0.56-1.22) | 0.83(0.56-1.22) | 2 | 0 | - | - | - |  | - | - |
|  | LOAD | Dominant | 3024/3014 | 1.09(0.95-1.26) | 1.08(0.96-1.20) | 11.46 | 30.2 | - | - | - |  | - | - |
|  |  | Recessive ^a^ | 3024/3014 | 0.93(0.74-1.15) | 0.92(0.76-1.11) | 9.4 | 14.9 | - | - | - |  | - | - |
| Female | Caucasian | Dominant | 1676/1619 | 1.18(1.01-1.37) | 1.18(1.01-1.37) | 7 | 0 | - | - | - | - | - | - |
|  |  | Recessive | 1676/1619 | 1.40(0.94-2.10) | 1.40(0.94-2.10) | 7 | 0 | 1550/1502 | 1.33(0.88-2.01) | 1.33(0.88-2.01) | 5 | 0 | [34,42] |
|  | Asian | Dominant | 1390/1315 | 1.18(0.91-1.52) | 1.15(0.98-1.36) | 13.11 | 46.6 | - | - | - | - | - | - |
|  |  | Recessive | 1390/1315 | 1.08(0.85-1.38) | 1.09(0.90-1.33) | 9.27 | 24.5 | - | - | - | - | - | - |
| Male | Caucasian | Dominant | 836/936 | 1.06(0.87-1.29) | 1.06(0.87-1.29) | 7 | 0 | - | - | - | - | - | - |
|  |  | Recessive | 836/936 | 1.10(0.66-1.83) | 1.11(0.68-1.82) | 7.19 | 2.7 | 595/668 | 1.11(0.65-1.91) | 1.11(0.65-1.91) | 5 | 0 | [34,41] |
|  | Asian | Dominant | 897/943 | 0.96(0.79-1.18) | 0.96(0.79-1.18) | 7 | 0 | - | - | - | - | - | - |
|  |  | Recessive | 897/943 | 0.91(0.63-1.30) | 0.88(0.70-1.12) | 14.49 | 51.7 | - | - | - | - | - | - |
| LOAD | Female | Dominant | 1383/1211 | 1.23(1.03-1.46) | 1.23(1.03-1.46) | 5 | 0 | - | - | - | - | - | - |
|  |  | Recessive ^a^ | 1337/1178 | 1.47(0.88-2.44) | 1.47(0.88-2.44) | 4 | 0 | 1269/1122 | 1.42(0.83-2.43) | 1.42(0.85-2.38) | 3.22 | 6.7 | [34] |
|  | Male | Dominant | 682/666 | 1.10(0.88-1.39) | 1.10(0.88-1.39) | 5 | 0 |  | - | - | - | - | - |
|  |  | Recessive ^a^ | 664/654 | 0.85(0.30-2.25) | 1.06(0.56-2.02) | 7.13 | 43.9 | * - | - | - | - | - | - |

a One study about African (Desaia et al)for recessive model was not sufficient to calculated pooled OR.

* Pooled ORs were not calculated for only two articles left sufficient to calculate pooled ORs after excluding articles with OR>3.0 or OR<0.3.

Dominant model, AA+GA vs. GG; Recessive model, AA vs. GA+GG.

FEM, fixed-effects model; REM, random-effects model. EOAD, early-onset Alzheimer's Disease; LOAD, late-oneset Alzheimer's Disease

| **Table S3.** Subgroup analysis on the relation of BDNF gene rs2030324 polymorphism with AD in dominant and recessive model | | | | | | | | | | | | | |
| --- | --- | --- | --- | --- | --- | --- | --- | --- | --- | --- | --- | --- | --- |
| Data | Type | Inherited model | All included articles | | | | | After excluding articles with OR>3.0 or OR<0.3 | | | | | |
|  |  |  | Number | REM Pooled OR (95% CI) | FEM Pooled OR (95% CI) | Q-value | I² (%) | Number | REM Pooled OR (95% CI) | FEM Pooled OR (95% CI) | Q-value | I² (%) | Articles Excluded |
| Ethnicity | Caucasian | Dominant | 3674/3355 | 0.97(0.78-1.22) | 1.01(0.88-1.16) | 28.82 | 54.9 | 3462/3153 | 0.93(0.78-1.10) | 0.95(0.83-1.10) | 16.00 | 25 | [48] |
|  |  | Recessive ^a^ | 3312/3088 | 1.10(0.86-1.40) | 1.10(0.86-1.40) | 11.00 | 0 | 2448/2380 | 1.06(0.83-1.36) | 1.06(0.83-1.36) | 10.00 | 0 | [31,36,48] |
|  | Asian | Dominant | 2064/2309 | 1.24(0.82-1.89) | 1.24(0.99-1.56) | 20.23 | 65.4 | 1789/1706 | 1.14(0.85-1.53) | 1.12(0.88-1.44) | 6.61 | 24.4 | [25,51] |
|  |  | Recessive ^b^ | 1107/1317 | 0.81(0.17-3.88) | 0.81(0.18-3.71) | 3.17 | 5.3 | * - | - | - | - | - | - |
| Form | EOAD | Dominant | 303/1359 | 1.38(0.65-2.91) | 1.52(0.92-2.52) | 6.16 | 51.3 | - | - | - | - | - | - |
|  |  | Recessive ^c^ | - | - | - | - | - | - | - | - | - | - | - |
|  | LOAD | Dominant | 2038/2351 | 1.20(0.77-1.88) | 1.16(0.95-1.42) | 33.61 | 76.2 | 1760/1651 | 0.90(0.72-1.12) | 0.90(0.72-1.12) | 6.00 | 0 | [48,51] |
|  |  | Recessive ^d^ | 2038/2351 | 2.59(0.54-12.40) | 2.64(0.66-10.53) | 5.11 | 21.7 | *- | - | - | - | - | - |
| Sex | Female | Dominant | 696/603 | 1.07(0.76-1.49) | 1.07(0.76-1.49) | 2.00 | 0 | - | - | - | - | - | - |
|  |  | Recessive ^c^ | - | - | - | - | - | - | - | - | - | - | - |
|  | Male | Dominant | 356/444 | 0.87(0.56-1.34) | 0.87(0.56-1.34) | 2.00 | 0 |  | - | - |  | - | - |
|  |  | Recessive ^c^ | - | - | - |  | - | - | - | - | - | - | - |

a Two studies(Lee et al. Bodner et al) for recessive model were not sufficient to calculated pooled OR.

b Four studies(Hou et al.Akatsu et al.Matsushita et al.Masataka et al.) for recessive model were not sufficient to calculated pooled OR.

c Pooled ORs were not calculated for less than two articles left sufficient to calculate pooled ORs for recessive model.

d Four studies(Hou et al.Akatsu et al.Matsushita et al.Riemenschneider et al.) for recessive model were not sufficient to calculated pooled OR.

* Pooled ORs were not calculated for only two articles left sufficient to calculate pooled ORs after excluding articles with OR>3.0 or OR<0.3

FEM, fixed effect model; REM, random effect model. EOAD, early-onset Alzheimer's disease; LOAD, late-onset Alzheimer's disease

Dominant model, TT+CT vs. CC; Recessive model, TT vs. CT+CC.
